# Comparison of Four ChIP-Seq Analytical Algorithms Using Rice Endosperm H3K27 Trimethylation Profiling Data

**DOI:** 10.1371/journal.pone.0025260

**Published:** 2011-09-30

**Authors:** Brandon M. Malone, Feng Tan, Susan M. Bridges, Zhaohua Peng

**Affiliations:** 1 Department of Computer Science and Engineering, Mississippi State University, Mississippi, United States of America; 2 Institute for Genomics, Biocomputing, and Biotechnology, Mississippi State University, Mississippi, United States of America; 3 Department of Biochemistry and Molecular Biology, Mississippi State University, Mississippi, United States of America; Michigan State University, United States of America

## Abstract

Chromatin immunoprecipitation coupled with high throughput DNA Sequencing (ChIP-Seq) has emerged as a powerful tool for genome wide profiling of the binding sites of proteins associated with DNA such as histones and transcription factors. However, no peak calling program has gained consensus acceptance by the scientific community as the preferred tool for ChIP-Seq data analysis. Analyzing the large data sets generated by ChIP-Seq studies remains highly challenging for most molecular biology laboratories.

Here we profile H3K27me3 enrichment sites in rice young endosperm using the ChIP-Seq approach and analyze the data using four peak calling algorithms (FindPeaks, PeakSeq, USeq, and MACS). Comparison of the four algorithms reveals that these programs produce very different peaks in terms of peak size, number, and position relative to genes. We verify the peak predictions using ChIP-PCR to evaluate the accuracy of peak prediction of the four algorithms. We discuss the approach of each algorithm and compare similarities and differences in the results. Despite their differences in the peaks identified, all of the programs reach similar conclusions about the effect of H3K27me3 on gene expression. Its presence either upstream or downstream of a gene is predominately associated with repression of the gene. Additionally, GO analysis finds that a substantially higher ratio of genes associated with H3K27me3 were involved in multicellular organism development, signal transduction, response to external and endogenous stimuli, and secondary metabolic pathways than the rest of the rice genome.

## Introduction

Chromatin immunoprecipitation (ChIP) coupled with high throughput sequencing (ChIP-Seq) has emerged as one of the most promising tools for profiling protein-DNA binding sites and chromatin modifications on a genome-wide scale [1]. The goal of ChIP-Seq studies is to find those genomic DNA fragments that are enriched in immunoprecipitation fractions using antibodies specific for DNA associated proteins of interest. Enriched regions, those with a high density of short DNA reads after immunoprecipitation and DNA sequencing, are referred to as peaks. Many programs for identification of peaks with ChIP-Seq data have been developed in recent years [2], [3], [4], [5], [6], [7], [8], [9], [10], [11]. The reported algorithms differ in their approaches for identifying potential enriched regions of the genome. Some algorithms, for example MACS [10] and PeakSeq [8], use a simple sliding window and group all reads within each window together. Others use a finer resolution method, either considering each base pair singly as in FindPeaks [4] or defining the windows based on the read locations as represented by USeq [7]. After identifying windows, the algorithms must then determine which windows are the true enriched regions. Methods without a control (FindPeaks) either simply report the number of reads in the windows or make an assumption about the background distribution, such as assuming the reads follow a Poisson distribution (FindPeaks), and calculate significance based on the assumed distribution. Those including a control sample (MACS, PeakSeq) use the control to more accurately model the background distribution of the reads and calculate an empirical False Discovery Rate (FDR) via, for example, a sample swap technique. Distinguishing between multiple small peaks or a single large peak is also challenging. While some algorithms merge overlapping peaks (MACS) or peaks within a user-supplied threshold (USeq, PeakSeq), others (FindPeaks) compare the height of peaks to the depth of the separating valley to differentiate multiple small peaks from one large peak. Pepke *et al.* [12] discussed a number of additional peak identification algorithms in a review article. They made distinctions among the algorithms, including how the algorithms aggregated the reads, the criteria for significant peak identification, read shifting to account for reading the end of the reads, use of control, and input parameters. Similarly, Barski and Zhao [13] also reviewed a number of algorithms for peak identification. Thus far, however, no program has emerged as the consensus best approach for identifying peaks in histone modification and DNA binding studies. Therefore, it is important to compare these available algorithms and to suggest essential parameters to assist molecular biology laboratories in selecting the best program for their data analysis.

Previous studies have shown that the repressive function of histone 3 modification H3K27me3 is conserved between plants and animals although the modification patterns and the mechanisms by which H3K27me3 is established or maintained may be different [14], [15]. In animals, H3K27me3 is established and maintained by polycomb-group (PcG) protein complexes *PhoRC*, *PRC1* and *PRC2* [16], [17]. These complexes repress selected genes at appropriate developmental stages [17], [18]. Several critical imprinted genes in *Arabidopsis* endosperm have been shown to be associated with H3K27me3. The FIS class gene products, MEA, FIS2, and FIE appear to function in a large PcG complex along with additional components such as MULTI-COPY SUPPRESSOR OF IRA1 (MSI1) and retinoblastoma-related protein RBR1 [19]. The PcG complex is predicted to repress gene transcription via histone H3K27 trimethylation and chromatin remodeling, and the established patterns are stably propagated through mitotic cell cycles [20]. MEA itself is imprinted via H3K27me3 [21], [22]. The self-imprinting mechanism of MEA, in which maternally expressed MEA replenishes the FIS-PcG complex, and in turn, the complex keeps repressing the silenced paternal MEA allele [22], [23]. PHERES1 (PHE1) is another imprinted gene in the *Arabidopsis* endosperm. The silenced maternal PHE1 allele is a direct target of the FIS-PcG complex [24]. Histone H3K27 trimethylation via the FIS-PcG complex likely both establishes and maintains the silencing of the paternal PHE1 [24], [25]. Zhang *et al*. used ChIP-chip to reveal that H3K27me3 regulates an unexpectedly large number of genes (∼4,400), including numerous transcription factors in *Arabidopsis* young seedlings [14]. The H3K27me3 profile has also been examined in rice seedlings [26]. However, the DNA binding profile and the function of H3K27me3 in plant endosperm remain unknown. A genome wide profiling of the enrichment sites of H3K27me3 in endosperm using ChIP-Seq will provide critical insight into the specific role of H3K27me3 modification in endosperm. In addition, analysis of the data set using multiple analytical algorithms will provide an unbiased comparison of these analytical algorithms.

In this report, we identified H3K27me3 modification sites within rice (*Oryza sativa*) young endosperm using the ChIP-Seq approach. Four different peak identification algorithms (PeakSeq, USeq, MACS, and FindPeaks) were used to locate H3K27me3 enrichment sites. ChIP-PCR was used to evaluate the quality of the peaks identified by these algorithms. We also analyzed the relative location of the peaks with respect to gene expression. Finally, we examined the Gene Ontology (GO) annotations [27] of the ChIP enriched genes.

## Results

### Chromatin Immunnoprecipitation, DNA Sequencing, and Mapping the Short Reads to the Genome

Chromatin was isolated from young rice endosperm and fragmented to a size range from 150 to 400 bp. The solubilized chromatin fragments were immunoprecipitated with antibodies against H3K27me3 (Millipore). The recovered DNA fragments were processed for DNA sequencing by Illumina. The reads produced by the Illumina Genome Analyzer were 36 base pairs long. Two DNA samples were analyzed. One was immunoprecipitated DNA samples enriched for H3K27me3, while the other was a control using sonicated genomic DNA fragments. A total of 10,999,931 ChIP reads were produced, and SeqMap [28] uniquely mapped 9,554,767 (∼85%) to the TIGR 6 rice genome [29]. A total of 11,986,448 control reads were produced, and 7,202,808 (∼60%) uniquely mapped to the genome. A larger percentage of the ChIP reads mapped uniquely. This was probably because immunoprecipitated reads were found more frequently associated with genic regions than the control reads. In particular, 31.9% (p-value<2.2e-16) of the H3K27me3 reads mapped to genic regions. In contrast, only 23.9% (p-value 1) of the input DNA mapped to genic regions. The p-values were calculated using a binomial test. These results are consistent with previous ChIP-chip report in plants [14].

### Peak Identification

**Figure 1:**
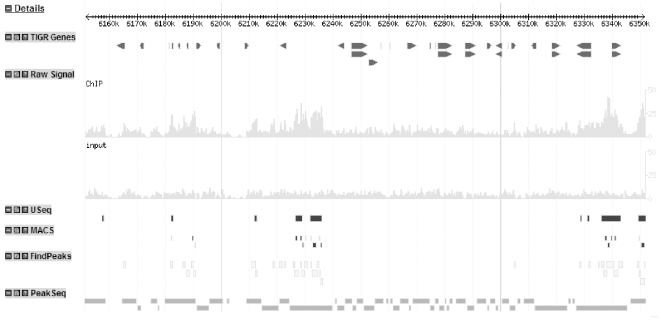
GBrowse visualization of identified peaks. This image displays a 200 kb region of rice chromosome 1. The top track indicates the positions of all genes identified by TIGR, v6. The next two tracks display the distribution of ChIP and Input (control) reads respectively. The following four tracks display the predicted peaks by each of the four examined peak calling programs. The sequence read numbers were normalized to ensure that the ChIP and the Input had identical read numbers over the total genome. Therefore, the height of the graph in this figure directly correlates with the read number in the region to visually display the DNA enrichment.

Four peak calling programs (MACS [10], PeakSeq [8], FindPeaks version 3.1.9 [4], and USeq [7]) were used to identify peaks of the mapped reads, respectively. Table 1 summarizes characteristics of the peaks identified by each of the programs and Figure 1 shows an example of some of the peaks identified by the different programs displayed in GBrowse [30].

**Table 1:** Statistical comparison of peaks identified by different peak calling programs.

Program	Peak Count	Base pair coverage	Percent coverage	Mean peak bandwidth	Standard deviation
PeakSeq, 200	71,269	170,523,735	43.8%	2392.7	3518.7
PeakSeq, 350	43,343	213,874,615	55.0%	4934.5	6805.2
PeakSeq, 589	23,760	268,102,711	68.9%	11283.8	14890.5
FindPeaks	41,516	35,140,770	9.0%	846.4	429.7
USeq	9,094	21,031,355	5.4%	2312.7	3176.2
MACS	15,738	12, 227,095	3.1%	777.9	831.5

This table shows some basic statistics about the peaks identified by each of the peak calling programs. The percent coverage indicates the percentage of the genome identified as part of a peak. To compute the percentage, a genome size of 389 Mb was used [38]. The PeakSeq program was run three times with a different max_gap parameter each time. The number following the comma indicates the value used for the max_gap.

The results show that the average peak bandwidth called by PeakSeq ranges from 2,393 bp to 11,284 bp depending on the max_gap parameter. Smaller values of the max_gap parameter result in shorter peak bandwidths. In the remainder of this manuscript, all PeakSeq results use max_gap = 200 and the program is referred to as PeakSeq(200). The peaks produced by FindPeaks have an average bandwidth of 846 bp while those identified by Useq average 2,313 bp—similar to those identified by PeakSeq(200). The peak bandwidth identified by MACS is the shortest at 778 bp on average.

FindPeaks identified 41,516 peaks covering 9.0% of the genome, USeq identified 9,094 peaks covering 5.4% of the genome, and MACS identified 15,738 peaks covering 3.1% of the genome. In contrast, peaks identified by PeakSeq with different max_gap values covers from 44% to 68.9% of the genome and identifies from 23,760 to 71,269 peaks.

The identified peaks were further characterized by their frequency of being identified by the four programs and the variation in peak height for each program (Table 2). All the peaks identified by MACS (100%) and 99% of the peaks identified by USeq are also identified by at least one other program. In contrast, 43.19% of the peaks identified by PeakSeq are not identified by any other program. Visual inspection of the ChIP and Input reads in GBrowse shows that many peaks identified by PeakSeq occur in regions where no substantial differences between the Input and the ChIP can be observed. Therefore we conclude that many of the peaks identified by PeakSeq are false positives. This is further indicated by the lowest average peak height (∼10.51) for PeakSeq among the four algorithms (Table 2). FindPeaks and USeq peaks have similar average peak heights (∼17.85 and ∼17.87, respectively) while those of MACS (∼14.05) are lower than these two. USeq combines many small peaks into a single peak resulting in a larger average peak bandwidth than FindPeaks or MACS. Although FindPeaks does not make use of the Input (control) data, most of the peaks it identifies are confirmed by other programs.

**Table 2:** Comparison of peak characteristics of different peak calling programs.

Program	One Support	Two Support	Three Support	Four Support	Peak Height
PeakSeq	43.19%	23.27%	15.84%	17.71%	10.51
FindPeaks	0.94%	37.32%	20.79%	40.96%	17.85
USeq	0.01%	16.05%	38.01%	45.92%	17.87
MACS	0.00%	40.95%	27.87%	31.18%	14.05

Table entries show the overlap of peaks identified by each program with those identified by other programs. Each column entry gives the percent of base pairs belonging to peaks identified by the specified program that were also identified as in peaks by other programs. Peak height gives the mean difference between the highest and lowest read counts of the peaks identified by that program.

### The Correlation of H3K27me3 Peaks and Gene Expression

To correlate gene expression with the H3K27me3 peaks, we carried out a gene expression profile study using an Affymetrix rice whole genome array with the cDNAs of rice endosperm at the same developmental stage as that in the ChIP experiment. TIGR 6 identifies 73,403 genes (including some alternate splice forms), but only 35,522 genes were represented on the gene expression microarray. In order to investigate the relationship between gene expression and ChIP-Seq peaks, only those genes with gene expression values were included in the analysis. These genes were grouped into four categories based on the location of H3K27me3 peaks relative to the gene: “within” the gene, “upstream” of the gene less than 2 kb, “downstream” of the gene less than 2 kb, and “none” within 2 kb either downstream or upstream of the gene. In some cases, a gene could be assigned to multiple categories. We found that this involved a relatively small percentage of the genes (about 10.47% with USeq) and therefore concluded that assigning each gene to a single category would not significantly alter the analysis results.

**Figure 2:**
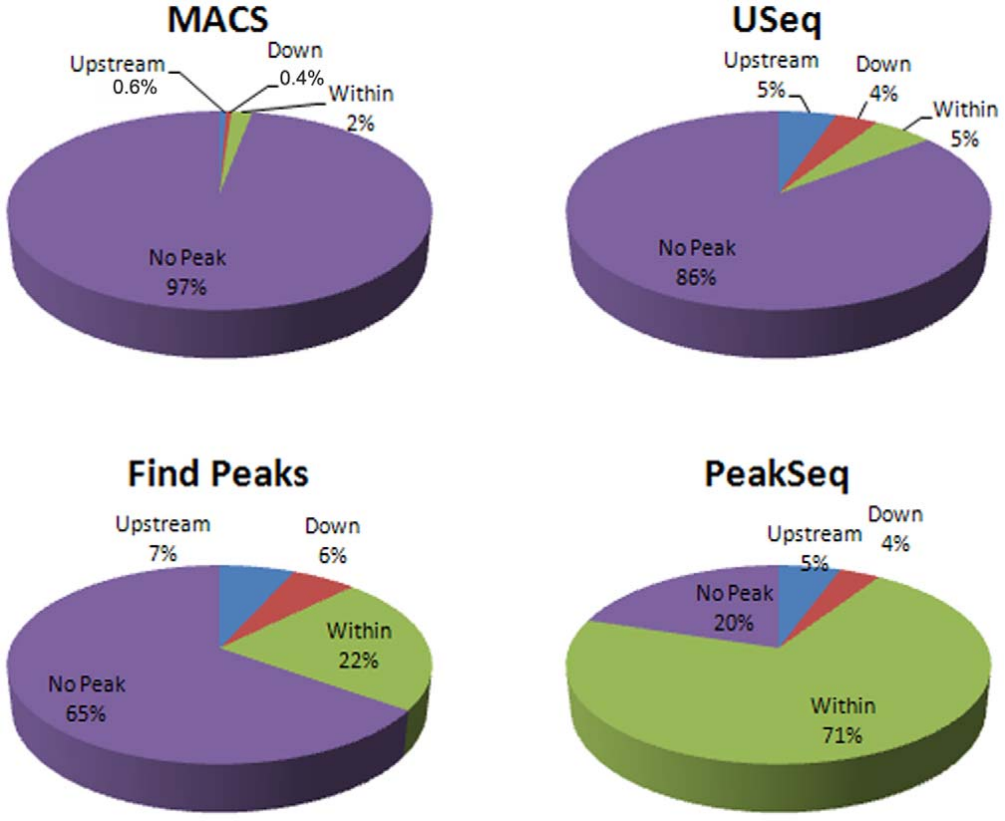
Distribution of gene classifications. These pie charts detail the portion of genes falling into each of the four categories based on their position relative to ChIP-Seq peaks: Upstream, Downstream, Within and No peak. The label above each pie chart indicates the program with which the pie chart is associated. In all cases, the blue portion represents the genes with an upstream peak, the red represents genes with a downstream peak, the green represents genes with a peak within the gene, and the purple represents genes with no peak identified.

Figure 2 displays the distribution of peaks identified by each of the peak identification programs. It is clear that the peaks identified by different programs displayed substantial differences. MACS, USeq, FindPeaks and PeakSeq(200) identified 97%, 86%, 65% and 20% of the total genes with no peak, respectively. For H3K27me3 peaks identified to be within a gene, the ratio was 2% for MACS, 5% for USeq, 22% for FindPeaks, and 71% for PeakSeq. For H3K27me3 peaks in the promoter region, the ratio was about 0.6% for MACS, 5% for USeq, 7% for FindPeaks, and 5% for PeakSeq. For the H3K27me3 peaks downstream of a gene, the ratio was about 0.4% for MACS, 4% for USeq, 6% for FindPeaks and 4% for PeakSeq, respectively. These results demonstrate that different programs will identify different peaks although the same dataset is used.

**Figure 3:**
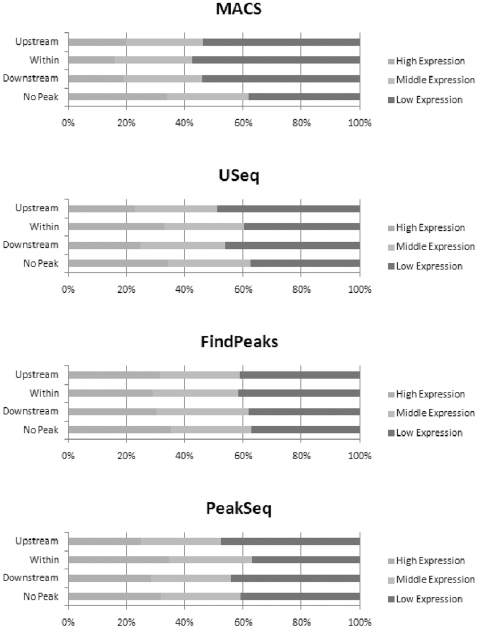
Conditional probabilities of gene expression versus peak classification. This figure illustrates the conditional probabilities of gene expression versus peak classification for each of the four peak identification programs. For example, the top bar for MACS indicates that approximately 20% of the genes with a peak upstream had high expression, while about 50% of those with a peak upstream had low gene expression.

The expression values were discretized into three categories: high, middle, and low as described in Materials and Methods. We categorize all genes according to computed expression level, with 12,093 genes in high group, 10,204 genes in middle group, and 13,944 genes in low group, which are 33%, 28% and 39% of the total microarray identified genes, respectively. The number of genes in each of the categories is similar. We further examined the conditional probability of gene expression and peak category using the three gene expression classifications and the four peak categories described above. Figure 3 shows the percentage of genes in the three expression categories for the four types of peaks identified by each of the peak calling programs, respectively. Table 3 shows the significance of the relationships between gene expression and the peaks identified by each program. The p-values were computed using a hypergeometric test and represent the probability of obtaining each of the conditional probability values by chance. Despite the wide variation in the peak characteristics, the peaks from all of the programs result in similar biological conclusions. As Table 3 indicates, regardless of the program used to identify the peaks, a statistically significant relationship exists between the probability of low gene expression and an upstream H3K27me3 peak. Furthermore, all of the programs except FindPeaks indicate a significant relationship between downstream peaks and low gene expression. These results suggest that the presence of H3K27me3 upstream or downstream of a gene is frequently associated with transcriptional repression. Similar conclusions were reached in ChIP-chip studies in *Arabidopsis* [14] and ChIP-Seq studies in rice young seedlings [26].

**Table 3:** p-values for associations between gene expression and gene classification.

Expression Value	Peak Location	FindPeaks	PeakSeq	USeq	MACS
High	Downstream	1.00	1.00	1.00	1.00
High	None	**0.00**	1.00	**0.00**	**0.00**
High	Upstream	0.98	1.00	1.00	1.00
High	Within	1.00	**0.00**	0.66	1.00
Low	Downstream	0.65	**0.00**	**0.00**	**0.00**
Low	None	1.00	**0.00**	1.00	1.00
Low	Upstream	**0.00**	**0.00**	**0.00**	**0.00**
Low	Within	**0.00**	1.00	0.17	**0.00**
Middle	Downstream	**0.00**	0.73	0.22	0.59
Middle	None	1.00	0.94	0.52	0.13
Middle	Upstream	0.72	0.84	0.47	0.67
Middle	Within	0.02	0.02	0.70	0.80

This table shows the results of using a hypergeometric test to test for statistical significance between the expression value for genes and the peak classification for genes. The expression value and peak location indicate the classification of the genes for expression and peak classification, respectively. The remaining four columns provide the calculated p-values based on peak classifications for each of the peak identification programs. The statistically significant cells are bold. A cut-off of 10^−2^ was used to identify significant relationships.

### Verifying Peak Predictions by ChIP-PCR

**Figure 4:**
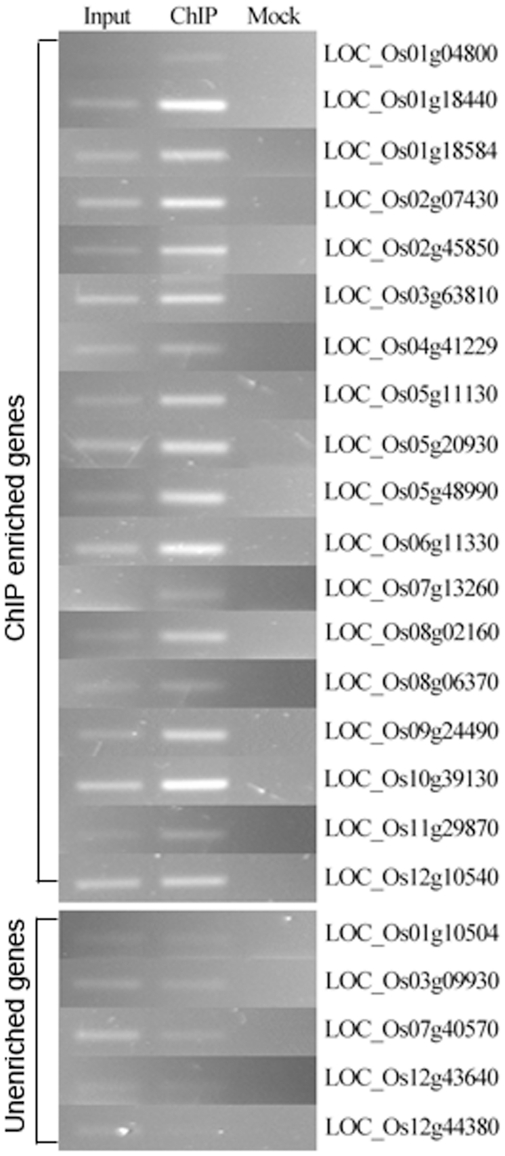
Semiquantitative PCR analyses of Regions Enriched by ChIP. Antibodies for H3K27me3 were used for ChIP experiments with the chromatin isolated from rice endosperm. **Input:** DNA sample extracted from the chromatin before the chromatin immunoprecipitation step. **ChIP:** DNA sample extracted from ChIP enriched chromatin. **Mock:** DNA extracted from sample that went through the IP procedure without antibody. Primers were designed to amplify the gene indicated on the right. The amplified fragment sizes were from 100 bp to 260 bp. About 20 ng of template DNA were used and the DNA was amplified for twenty four thermal cycles. The genes were selected based on ChIP-Seq data analysis results, including eighteen enriched and five unenriched genes as indicated in the figure.

In order to further evaluate the peak algorithm results, we selected 18 genes with H3K27me3 peaks and 5 genes without peaks identified visually on the genome browser according to the sequence reads profile. Then we performed ChIP followed by semi-quantitative PCR experiments for these 23 genes. Figure 4 shows the results of the ChIP-PCR experiments. The eighteen genes with enriched peaks showed the same enrichment pattern using ChIP-PCR. We further checked the classification of these genes by each of the peak identification algorithms. The results revealed that FindPeaks, MACS, PeakSeq and USeq successfully classify 13, 2, 18 and 12 of the 18 enriched genes, respectively (Table 4). Only PeakSeq classifies one of the five unenriched genes as enriched. These results suggest that although PeakSeq successfully identifies the genes with enrichment (high sensitivity), its specificity suffers when compared to the other programs. On the other hand, although MACS generates high resolution peaks, the sensitivity in peak identification is compromised. The USeq also lost some peaks presented in the cell.

**Table 4:** Algorithm classifications for PCR genes.

Gene	PCR Result	FindPeaks	MACS	PeakSeq	USeq
LOC_Os01g04800	+	+	−	+	+
LOC_Os01g18440	+	+	−	+	+
LOC_Os01g18584	+	+	−	+	+
LOC_Os02g07430	+	+	−	+	+
LOC_Os02g45850	+	+	−	+	+
LOC_Os03g63810	+	+	−	+	−
LOC_Os04g41229	+	−	−	+	−
LOC_Os05g11130	+	+	−	+	+
LOC_Os05g20930	+	−	−	+	−
LOC_Os05g48990	+	−	−	+	−
LOC_Os06g11330	+	+	−	+	+
LOC_Os07g13260	+	+	−	+	+
LOC_Os08g02160	+	−	−	+	−
LOC_Os08g06370	+	+	−	+	+
LOC_Os09g24490	+	+	−	+	+
LOC_Os10g39130	+	−	−	+	−
LOC_Os11g29870	+	+	+	+	+
LOC_Os12g10540	+	+	+	+	+
LOC_Os03g09930	−	−	−	−	−
LOC_Os01g10504	−	−	−	−	−
LOC_Os12g43640	−	−	−	−	−
LOC_Os12g44380	−	−	−	−	−
LOC_Os07g40570	−	−	−	+	−

This table shows the result of the PCR analysis. The first column indicates the TIGR rice gene name. The second column shows the PCR result. The remaining columns indicate the classification of the gene by each of the programs. A “+” indicates a ChIP-enriched gene, while a “−” indicates an unenriched gene.

### GO Annotations of H3K27me3 Associated Genes

**Figure 5:**
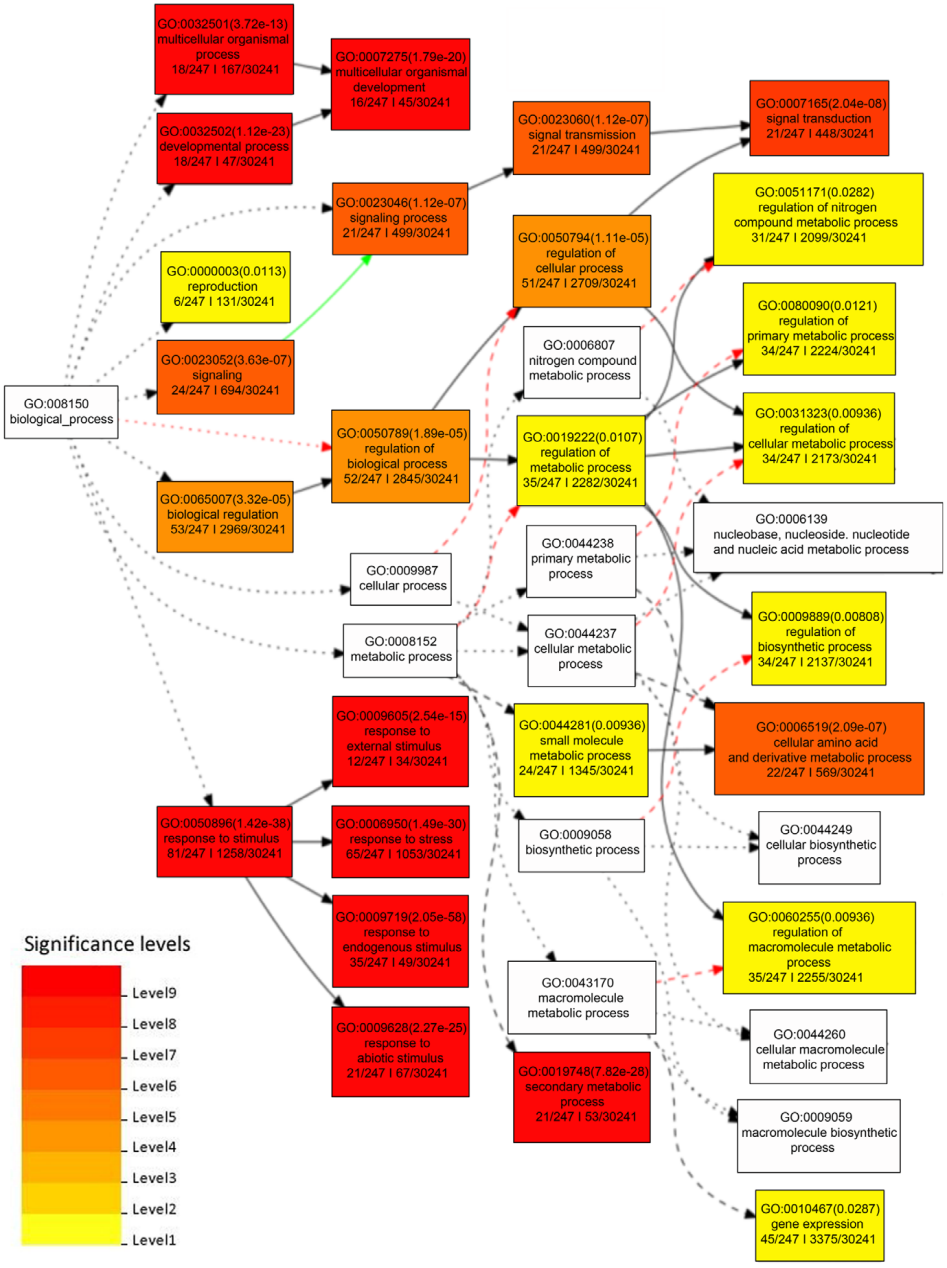
Enriched GO biological processes of genes with low expression and an upstream peak. This figure shows the significant biological process GO annotations for genes with low gene expression and a MACS peak upstream. The top line in each of the boxes lists the GO identifier of the term and the statistical significance (multiple hypothesis corrected p-value, lower is more significant) of that annotation. The middle lines are a description of the GO term. The four numbers on the bottom line are the number of genes with low expression and an upstream peak that had the annotation, the number of genes with low expression and an upstream peak that had any annotation (always 247), the total number of genes that had the annotation and the total number of genes that had any annotation (always 30241). The color of the box is an indication of the significance of the term. White boxes are not significant. The higher level it is, the more significant the GO term is. The color of the arrows indicates the relationship among the GO terms. Black signifies “is_a.” Orange is “part_of;” red is “positive_regulate.” Purple is “regulate,” while green is “negative_regulate.” Long dashes indicate “two significant nodes,” and short dashes mean “one significant node.”

**Figure 6:**
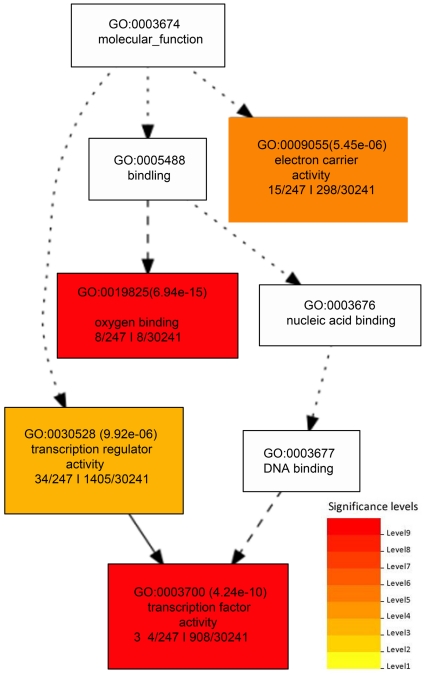
Enriched GO molecular functions of genes with low expression and an upstream peak. This figure shows the significant molecular function GO annotations for genes with low gene expression and a MACS peak upstream. The notation and coloring are the same as that described in Figure 5.

**Figure 7:**
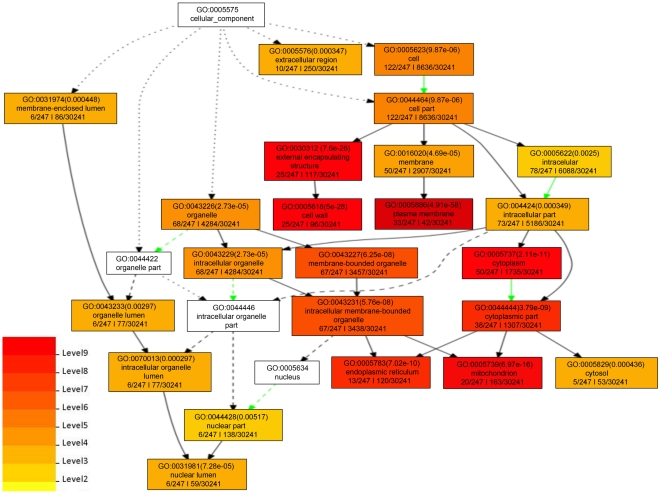
Enriched GO cellular components of genes with low expression and an upstream peak. This figure shows the significant cellular component GO annotations for genes with low gene expression and a MACS peak upstream. The notation and coloring are the same as that described in Figure 5.

We next analyzed the GO annotations assigned to the genes with significant enrichment. The GO is divided into three distinct categories: (i) the biological processes (BP) in which the gene product participates; (ii) the molecular functions (MF) that describe the gene product activities, such as catalytic or binding activities, at the molecular level; and (iii) the cellular component (CC) where the gene product can be found. We used agriGO [31] to identify GO annotations for which each of the groups of genes were significantly enriched compared to all genes in the genome. Because MACS' peaks were supported by all four programs, we selected those for GO analysis. Figures 5, 6 and 7 show the enriched GO annotations for genes with low expression and upstream peaks in the biological process, molecular function and cellular component categories, respectively. H3K27me3 seems to particularly affect multicellular organism processes and development, signal transduction, response to stimuli (including external stimulus, endogenous stimulus and stress), and secondary metabolic process as shown in Figure 5. The biological processes tightly associated with H3K27me3 are transcription factor activities and oxygen binding (Figure 6). Furthermore, substantially more H3K27me3 modified genes encode proteins located in the cell wall, plasma membrane, ER, and mitochondrion (Figure 7). We found that a large number of cell wall metabolic pathway genes subjected to the regulation of H3K27me3 as shown in Table S1.

## Discussion

### Comparison of the four peak identification programs

We analyzed the performance of four different peak identification programs with ChIP-Seq data from rice endosperm. The programs produced quite different peaks in terms of peak size, number and relative position to a gene. We evaluated the peak predictions using ChIP-PCR and compared the accuracy of peak prediction of these algorithms. PeakSeq identifies a large number of peaks, which cover from 44% to 69% of the genome. However, the identified peaks were not very precise as shown in ChIP-PCR tests and in comparison of the read profiles between ChIP and Input samples in GBrowse. While MACS identified peaks were supported by other peak calling programs, this program might miss many true peaks as shown in our ChIP-PCR verification. USeq identified peaks are also reliable but the program only identifies large peaks. The smaller peaks were not detected or merged with nearby peaks. FindPeaks identified a large number of peaks with various sizes and the identified peaks were mostly reliable as judged by ChIP-PCR and the average peak height. However, FindPeaks does not take the control data into consideration while identifying the peaks. To acquire accurate peak calling results, the control data needs to be considered. A simple compensation method is to subtract the peaks generated by control data alone after normalization with the same program. A variety of differences in these algorithms, such as use of a control dataset and statistical corrections for read counts, can account for the differences. Given that each program has advantages and disadvantages, it is the best to analyze the data with multiple programs to verify the results. Meanwhile, it is still necessary to develop more ChIP-Seq data analysis tools to eventually identify a program that best fits the requirements of regular molecular biology laboratories for ChIP-Seq studies.

Deng's group published an H3K27me3 whole-genome profile from Nipponbare seedling shoots in the four-leaf stage [26]. Genomic regions associated with H3K27me3 modification were identified using MACS, in which the parameters (bandwidth, 300 bp; mfold, 32; p-value of 1.00e-05) were set up to call peaks representing enriched epigenetic marks. In their study, out of 41,043 non-TE genes, 17,211 (41.9%) were found to be modified by H3K27me3. Our study of an H3K27me3 whole genome profile in rice endosperm reveals that a total of 15,738 peaks were identified using MACS. The results in both seedlings and endosperm were similar although endosperm has fewer cell types.

### Function of H3K27 trimethylation

Despite the differences, all four of the ChIP-Seq data analysis programs reach similar conclusions about the effect of H3K27me3 on gene expression. Its presence upstream of a gene is often associated with repression of expression (Table 3 and Figure 3). Similarly, its presence downstream of a gene is also associated with repression of expression. Additionally, GO analysis revealed that many of the H3K27me3 associated genes were involved with multicellular organism development, signal transduction, response to external and endogenous stimuli, and secondary metabolic processes. Interestingly, cell wall related genes stand out as a distinct group that is regulated by H3K27 trimethylation. Detailed examination of the genes used for GO analysis shows that 25 out of 247 (10.1%) genes subjected to H3K27me3 regulation are involved in cell wall metabolism. The p value for such a distribution is 5E-28. Our results suggest that H3K27 trimethylation not only plays a key role in regulating development, signal transduction, and response to external and endogenous stimuli, but also is a key regulator of metabolism.

## Materials and Methods

### Plant materials

All plants used in this study were rice strain *Oryza sativa* ssp *japonica* cv Nipponbare. The immature rice seeds were harvested 6–7 days after pollination. The cross-linking of the chromatin was achieved by vacuum infiltrating PBS (pH7.4) with 1% formaldehyde for 15 min at room temperature. The cross-linking reaction was stopped by adding glycine to a final concentration of 0.125 M and incubating for 5 min under vacuum. The tissues were rinsed 3 times with PBS.

### Chromatin extraction

The chromatin was extracted from endosperm following the protocol of Gendrel *et al* [32] with minor modification. Briefly, after removal of the embryo, the seeds were ground to a fine powder in liquid nitrogen and resuspended in chromatin isolation buffer I (0.4 M sucrose, 10 mM Tris-Cl pH 8.0, 10 mM MgCl_2_, 5 mM β–mercaptoethanol, 1 mM PMSF) followed by filtration of the slurry through 4 layers of cheesecloth and 2 layers of Miracloth and centrifugation at 12,000 g for 10 min. The pellet was washed in buffer II (0.25 M sucrose, 10 mM Tris-Cl pH8.0, 10 mM MgCl_2_, 1% Triton X-100, 5 mM β–mercaptoethanol, 1 mM PMSF) and centrifuged as above. The pellet was homogenized in buffer III (1.7 M sucrose, 10 mM Tris-Cl pH8.0, 2 mM MgCl_2_, 0.15% Triton X-100, 5 mM β–mercaptoethanol, 1 mM PMSF) and layered over an equal volume of buffer III. The pellet of chromatin was recovered by centrifugation at 27,000 g for 30 min.

### Chromatin immunoprecipitation

ChIP experiments were carried out as described by Gendrel et al [30]. Briefly, the chromatin pellet was resuspended in lysis buffer (50 mM Tris-Cl pH 8.0, 10 mM EDTA, 1% SDS) and fragmented to a size range of 150–400 bases with the Sonic Dismembrator (Fisher, model# 550). Solubilized chromatin was immunoprecipitated with antibodies against H3K27me3 (Millipore, Cat# 07-449). Antibody-chromatin complexes were pulled-down using protein A agarose/Salmon Sperm DNA, washed and then eluted. After cross-link reversal and proteinase K treatment, immunoprecipitated DNA was extracted with phenol-chloroform, ethanol precipitated, and treated with RNase. ChIP DNA was quantified using PicoGreen. Input DNA was extracted from the initial solubilized chromatin.

### Library Preparation and Solexa Sequencing

Input and ChIP samples were processed following Illumina's protocol from the ChIP DNA Sample Prep Kit. Briefly, 10 ng input and ChIP enriched DNA was subjected to end repair, addition of “A” bases to 3′ ends, ligation of adapters, agrose gel size selection for fragments with average size about 186 bp, and PCR amplification to produce a DNA library of adapter-modified fragments. DNA sequencing was carried out using the Illumina/Solexa Genome Analyzer sequencing system at a concentration of 2 to 4 pM. Cluster amplification, linearization, blocking and sequencing primer reagents were provided in the Solexa Cluster Amplification kits and were used according to the manufacturer's specifications.

### Mapping the short reads to the genome

The generated short reads were mapped onto the genome using SeqMap [28] allowing up to two mismatches between the short read and genome. The Illumina reads were aligned to TIGR version 6 of the rice genome [29]. The alignments were output in ELAND format [33]. Only reads which mapped uniquely to the genome were retained.

### Identifying peaks

Each of the four peak calling programs (MACS [10], PeakSeq [8], FindPeaks [4], and USeq [7]) was used to identify peaks with the mapped reads. Tables S2, S3, S4, S5 show the parameters used when running the four peak calling programs. As previously described, these algorithms address the peak identification problem differently. Whenever possible, though, parameters were selected in a manner to be consistent in all programs for the purpose of comparison.

All the ChIP-Seq data were deposit to NCBI's Gene Expression Omnibus (http://www.ncbi.nlm.nih.gov/geo/) with the deposition number GSE27048 for genome-wide maps of chromatin state in rice endosperm.

### Calculating the support value of a peak by different programs

The support value of a peak by different programs was calculated on a base-pair basis. We labeled each base pair with the programs that identified that base pair as belonging to a peak. We call the number of programs which identified a base pair as belonging to a peak the supporters of that base pair. Then, we counted the number of base pairs with supporters of four (so all programs identified that base pair as belonging to a peak), three, two, and one and listed these support programs with the peak. Finally, we calculated the percentage of base pairs for each support value for each program. For example, if one program identified a total of 200 base pairs as belonging to peaks and of those 200, 60 had a support value of 2, then 30% of the base pairs for that program had support 2.

### Calculating peak height

The height of peaks was calculated on a per-peak basis. We counted the number of ChIP-Seq reads mapping to each position in the peak. The minimum number of reads was subtracted from the maximum number of reads in the peak. We then calculated the mean of this difference over all peaks for each program.

### Gene expression experiments

The immature rice seeds were harvested 6–7 days after pollination. After removal of the embryo, the seeds were ground to a fine powder in liquid nitrogen and re-suspended in RNA extraction buffer (50 mM Tris-Cl, pH 8.0, 150 mM LiCl, 5 mM EDTA, pH 8.0, 1% SDS). The mixture was extracted twice with phenol-chloroform and once with chloroform. A five volume of TRIZOL was added to the aqueous phase and then extracted once with chloroform. The RNA was precipitated with isopropanol. After washing with 70% ethanol, the RNA was dissolved into DEPC-H_2_O. After digestion with RNase-free DNase, the RNA was quantified with the NanoDrop method and qualified with Agilent 2100 Bioanalyzer. A 5 ug of RNA was used as starting material for the microarray experiment. The cRNA probe was labeled and hybridized to the gene chips according to the manufacturer's instruction (Affymetrix). The raw microarray data was extracted from the chip images by using the Gene Chip Operating Software (Affymetrix). All the microarray data is MIAME compliant and the raw data has been deposited to NCBI's Gene Expression Omnibus (http://www.ncbi.nlm.nih.gov/geo/) with the deposition number GSE26840 for expression data from rice endosperm.

### Characterizing gene expression levels

Each raw expression score was log transformed, and then a z-score was computed for each gene:
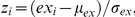
where 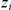 is the z-score for gene 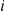, 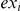 is the log base 10 expression value for gene 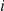, 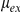 is the mean, and 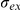 is the standard deviation. Note that the resulting z-scores have a mean of 0 and a standard deviation of 1.

The z-scores were then discretized into three categories: “high expression,” “middle expression” and “low expression” based on the number of standard deviations from the mean as shown below:
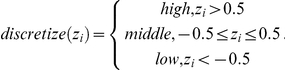



### Mapping between ChIP-Seq peaks and genes

The genes were partitioned into four disjoint sets based on where the peaks were located relative to the gene. If a gene body overlapped any peak, then the gene was labeled “within” (because the peak is within the gene). If any peak fell at most 2 kb upstream of a gene, it was labeled “upstream” (because the peak is upstream of the gene). Likewise, if any peak fell at most 2 kb downstream of a gene, it was labeled “downstream” (because the peak is downstream of the gene). Genes with no peaks within, upstream or downstream were labeled “none” or “no peak.” Each gene received only a single label. The precedence of the labels was “within,” “upstream,” “downstream” and “none.” This analysis was performed for each of the peak identification programs. We also tested using a window of 1 kb upstream of a gene and 500 bp downstream of a gene and obtained similar results.

### Computing conditional probabilities and statistical significance

Conditional probability is the probability that a proposition is true given that another proposition is true [34]. For example, 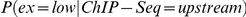 is the conditional probability that the gene expression for a particular gene is “low” given that the gene has an H3K27me3 peak “upstream.” Conditional probability can be calculated as:
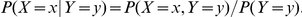
The conditional probabilities were calculated for gene expression given ChIP-Seq peak position relative to the gene. We computed p-values for the conditional probabilities using a hypergeometric test [35] implemented in R [36].

### Verifying peak predictions

In order to verify the peaks identified by the programs, we selected eighteen genes that were classified as having nearby peaks and five genes that were classified as having no nearby peaks based on the sequence read profile in genome browser for ChIP-PCR verification. Semi-quantification PCR reactions were performed for these genes; the primers used are listed in Table S6. The PCR reaction parameters were as follows: 1 cycle of 2 min at 95°C; 28 cycles of 30 s at 95°C, 30 s at 58°C, and 30 s at 72°C; cycle of 2 min at 75°C. The enrichment of H3K27me3 was determined by regular agarose gel electrophoresis.

### Investigating GO annotations of H3K27me3 enriched genes

Functional categorization of genes was carried out according to the GO rules [27] by agriGO [31]. Because of its high specificity, the gene classifications from MACS program were used. The query list for agriGO consisted of the genes which had an upstream peak and low gene expression, while the background was the TIGR gene model. Statistical significance was determined using a hypergeometric test using the Yekutieli multi-test adjustment [37].

### Data deposition

All the microarray data was MIAME compliant and that all the microarray and ChIP-Seq data were deposit to NCBI's Gene Expression Omnibus (http://www.ncbi.nlm.nih.gov/geo/) with the deposition number GSE26840 for expression data from rice endosperm and GSE27048 for genome-wide maps of chromatin state in rice endosperm.

## Supporting Information

Table S1 **Cell wall metabolic pathway genes subjected to the regulation of H3K27me3.**
(PDF)

Table S2 **FindPeaks program parameters.**
(PDF)

Table S3 **USeq program parameters.**
(PDF)

Table S4 **PeakSeq program parameters.**
(PDF)

Table S5 **MACS program parameters.**
(PDF)

Table S6 **The primers used in semi-quantification PCR reactions.**
(PDF)

## References

[1] Nix DA, Courdy SJ, Boucher KM (2008). Empirical methods for controlling false positives and estimating confidence in ChIP-Seq peaks.. BMC Bioinformatics.

[2] Barski A, Cuddapah S, Cui K, Roh T, Schones D (2007). High-resolution profiling of histone methylations in the human genome.. Cell.

[3] Boyle AP, Guinney J, Crawford GE, Furey TS (2008). F-Seq: a feature density estimator for high-throughput sequence tags.. Bioinformatics.

[4] Fejes AP, Robertson G, Bilenky M, Varhol R, Bainbridge M (2008). FindPeaks 3.1: a tool for identifying areas of enrichment from massively parallel short-read sequencing technology.. Bioinformatics.

[5] Kharchenko PV, Tolstorukov MY, Park PJ (2008). Design and analysis of ChIP-seq experiments for DNA-binding proteins.. Nat Biotechnol.

[6] Li H, Durbin R (2008).

[7] Nix DA, Courdy SJ, Boucher KM (2008). Empirical methods for controlling false positives and estimating confidence in chIP-seq peaks.. BMC Bioinformatics.

[8] Rozowsky J, Euskirchen G, Auerbach RK, Zhang ZD, Gibson T (2009). PeakSeq enables systematic scoring of ChIP-seq experiments relative to controls.. Nat Biotech.

[9] Schmid C, Bucher P (2007). ChIP-Seq Data Reveal Nucleosome Architecture of Human Promoters.. Cell.

[10] Zhang Y, Liu T, Meyer C, Eeckhoute J, Johnson D (2008). Model-based Analysis of ChIP-Seq (MACS).. Genome Biol.

[11] Zhang Y, Shin H, Song J, Lei Y, Liu XS (2008). Identifying Positioned Nucleosomes with Epigenetic Marks in Human from ChIP-Seq.. BMC Genomics.

[12] Pepke S, Wold B, Mortazavi A (2009). Computation for ChIP-seq and RNA-seq studies.. Nat Meth.

[13] Barski A, Zhao K (2009). Genomic Location Analysis by ChIP-Seq.. Journal of Cellular Biochemistry.

[14] Zhang X, Clarenz O, Cokus S, Bernatavichute YV, Pellegrini M (2007). Whole-genome analysis of histone H3 lysine 27 trimethylation in Arabidopsis.. PLoS Biol.

[15] Pien S, Grossniklaus U (2007). Polycomb group and trithorax group proteins in Arabidopsis.. Biochim Biophys Acta.

[16] Lee TI, Jenner RG, Boyer LA, Guenther MG, Levine SS (2006). Control of developmental regulators by Polycomb in human embryonic stem cells.. Cell.

[17] Boyer LA, Plath K, Zeitlinger J, Brambrink T, Medeiros LA (2006). Polycomb complexes repress developmental regulators in murine embryonic stem cells.. Nature.

[18] Tolhuis B, de Wit E, Muijrers I, Teunissen H, Talhout W (2006). Genome-wide profiling of PRC1 and PRC2 Polycomb chromatin binding in Drosophila melanogaster.. Nat Genet.

[19] Gehring M, Choi Y, Fischer RL (2004). Imprinting and seed development.. Plant Cell.

[20] Huh JH, Bauer MJ, Hsieh TF, Fischer R (2007). Endosperm gene imprinting and seed development.. Curr Opin Genet Dev.

[21] Gehring M, Huh JH, Hsieh TF, Penterman J, Choi Y (2006). DEMETER DNA glycosylase establishes MEDEA polycomb gene self-imprinting by allele-specific demethylation.. Cell.

[22] Jullien PE, Katz A, Oliva M, Ohad N, Berger F (2006). Polycomb group complexes self-regulate imprinting of the Polycomb group gene MEDEA in Arabidopsis.. Curr Biol.

[23] Baroux C, Gagliardini V, Page DR, Grossniklaus U (2006). Dynamic regulatory interactions of Polycomb group genes: MEDEA autoregulation is required for imprinted gene expression in Arabidopsis.. Genes Dev.

[24] Kohler C, Page DR, Gagliardini V, Grossniklaus U (2005). The Arabidopsis thaliana MEDEA Polycomb group protein controls expression of PHERES1 by parental imprinting.. Nat Genet.

[25] Makarevich G, Leroy O, Akinci U, Schubert D, Clarenz O (2006). Different Polycomb group complexes regulate common target genes in Arabidopsis.. EMBO Rep.

[26] He GM, Zhu XP, Elling AA, Chen LB, Wang XF (2010). Global Epigenetic and Transcriptional Trends among Two Rice Subspecies and Their Reciprocal Hybrids.. Plant Cell.

[27] Ashburner M, Ball CA, Blake JA, Botstein D, Butler H (2000). Gene ontology: tool for the unification of biology. The Gene Ontology Consortium.. Nature Genetics.

[28] Jiang H, Wong WH (2008). SeqMap: mapping massive amount of oligonucleotides to the genome.. Bioinformatics.

[29] Ouyang S, Zhu W, Hamilton J, Lin H, Campbell M (2007). The TIGR Rice Genome Annotation Resource: improvements and new features.. Nucleic Acids Res.

[30] Stein LD, Mungall C, Shu S, Caudy M, Mangone M (2002). The generic genome browser: a building block for a model organism system database.. Genome Res.

[31] Du Z, Zhou X, Ling Y, Zhang Z, Su Z (2010). agriGO: a GO analysis toolkit for the agricultural community.. Nucl Acids Res.

[32] Gendrel AV, Lippman Z, Martienssen R, Colot V (2005). Profiling histone modification patterns in plants using genomic tiling microarrays.. Nature Methods.

[33] Cox A (2001).

[34] Papoulis A (1984). Conditional Probabilities and Independent Sets. Probability, Random Variables, and Stochastic Processes.

[35] Feller W (1968). The Hypergeometric Series. An Introduction to Probability Theory and Its Applications. 3 ed.

[36] R Development Core Team (2006). R: A Language and Environment for Statistical Computing.

[37] Benjamini Y, Yekutieli D (2001). The control of the false discovery rate in multiple tesing under dependency.. Ann Stat.

[38] Sequencing ProjectInternational Rice G (2005). The map-based sequence of the rice genome.. Nature.

