# Supplementary material for: Comparison of Four ChIP-Seq Analytical Algorithms Using Rice Endosperm H3K27 Trimethylation Profiling Data
Source: PLoS One. 2011 Sep 30;6(9):e25260. doi: 10.1371/journal.pone.0025260 (PMC3184143; doi:10.1371/journal.pone.0025260)
Supplement: Table S1 — Cell wall metabolic pathway genes subjected to the regulation of H3K27me3. (PDF) [file pone.0025260.s001.pdf]

Table S1. Cell wall metabolic pathway genes subjected to the regulation of H3K27me3.

Locus ID	Gene Name
LOC_Os06g08380	1,3-beta-D-glucan synthase, putative
LOC_Os01g55040	1,3-beta-glucan synthase component, put
LOC_Os09g36180	68 kDa protein
LOC_Os08g14210	AJ555154 putative xyloglucan endotransg
LOC_Os02g16730	alpha-expansin
LOC_Os02g16780	alpha-expansin
LOC_Os02g16850	alpha-expansin
LOC_Os06g41700	alpha-expansin OsEXPA16
LOC_Os05g46200	At1g72990/F3N23_19
LOC_Os01g22550	At3g06440/F24P17_7
LOC_Os04g46740	AT5g47500/MNJ7_9
LOC_Os01g53750	AT5g56590/MIK19_3
LOC_Os04g44780	beta-expansin
LOC_Os10g40090	beta-expansin
LOC_Os03g01270	beta-expansin 3
LOC_Os10g40720	beta-expansin EXPB3
LOC_Os05g03840	cellulase (EC 3.2.1.4) At2g44540 - Arab
LOC_Os06g14540	cellulase (EC 3.2.1.4) F19H22.110 - Ara
LOC_Os09g36060	cellulase (EC 3.2.1.4) F19H22.110 - Ara
LOC_Os07g36610	Cellulose synthase
LOC_Os07g36630	Cellulose synthase
LOC_Os07g36690	Cellulose synthase
LOC_Os07g36700	Cellulose synthase
LOC_Os07g36750	Cellulose synthase
LOC_Os08g06380	Cellulose synthase
LOC_Os06g02180	Cellulose synthase, putative
LOC_Os05g08370	cellulose synthase-2
LOC_Os02g09930	cellulose synthase-like protein OsCslA9
LOC_Os07g36740	cellulose synthase-like protein OsCslF4
LOC_Os05g15880	chitinase (EC 3.2.1.14) III C00481 - ri
LOC_Os11g47570	chitinase (EC 3.2.1.14) III C00481 - ri
LOC_Os08g40740	chitinase (EC 3.2.1.14) III C10150 - ri
LOC_Os11g47550	chitinase (EC 3.2.1.14) III C10150 - ri
LOC_Os04g01930	contains similarity to limonene cyclase
LOC_Os04g21570	contains similarity to pollen surface p
LOC_Os09g25900	CSLC2
LOC_Os02g56740	eIF4-gamma/eIF5/eIF2-epsilon, putative
LOC_Os11g14400	endopolygalacturonase
LOC_Os03g05070	Exostosin family

LOC_Os01g59630	Exostosin family, putative
LOC_Os02g39960	Exostosin family, putative
LOC_Os03g01760	Exostosin family, putative
LOC_Os06g23420	Exostosin family, putative
LOC_Os06g46690	Exostosin family, putative
LOC_Os07g09050	Exostosin family, putative
LOC_Os07g37960	Exostosin family, putative
LOC_Os12g03100	Exostosin family, putative
LOC_Os12g12290	Exostosin family, putative
LOC_Os12g38450	Exostosin family, putative
LOC_Os02g26320	expressed protein
LOC_Os02g45700	expressed protein
LOC_Os02g49420	expressed protein
LOC_Os03g14130	expressed protein
LOC_Os03g20420	expressed protein
LOC_Os03g58070	expressed protein
LOC_Os06g44270	expressed protein
LOC_Os06g44660	expressed protein
LOC_Os07g07680	expressed protein
LOC_Os07g41650	expressed protein
LOC_Os08g04300	expressed protein
LOC_Os08g36840	expressed protein
LOC_Os10g05750	expressed protein
LOC_Os10g05950	expressed protein
LOC_Os10g05980	expressed protein
LOC_Os10g37260	expressed protein
LOC_Os10g39020	expressed protein
LOC_Os12g37660	expressed protein
LOC_Os04g42620	extensin-like protein
LOC_Os04g57430	extensin-like protein
LOC_Os12g03790	F21M12.27 gene product
LOC_Os01g47780	Fasciclin domain, putative
LOC_Os02g20540	Fasciclin domain, putative
LOC_Os02g20560	Fasciclin domain, putative
LOC_Os04g39590	Fasciclin domain, putative
LOC_Os06g17460	Fasciclin domain, putative
LOC_Os09g07350	Fasciclin domain, putative
LOC_Os09g30010	Fasciclin domain, putative
LOC_Os02g49140	galactosyl transferase GMA12/MNN10 fami
LOC_Os01g65590	Galactosyltransferase, putative
LOC_Os07g09670	Galactosyltransferase, putative
LOC_Os07g09690	Galactosyltransferase, putative
LOC_Os06g04620	GDP-mannose 4,6-dehydratase

LOC_Os07g13980	glucose-1-phosphate adenylyltransferase
LOC_Os01g04290	Glycosyl hydrolase family 10, putative
LOC_Os01g04300	Glycosyl hydrolase family 10, putative
LOC_Os02g50490	Glycosyl hydrolase family 9
LOC_Os02g53820	Glycosyl hydrolase family 9
LOC_Os06g13830	Glycosyl hydrolase family 9
LOC_Os08g02220	Glycosyl hydrolase family 9
LOC_Os02g03550	Glycosyl hydrolases family 16, putative
LOC_Os02g57770	Glycosyl hydrolases family 16, putative
LOC_Os07g34580	Glycosyl hydrolases family 16, putative
LOC_Os01g71410	Glycosyl hydrolases family 17
LOC_Os02g33000	Glycosyl hydrolases family 17
LOC_Os09g36280	Glycosyl hydrolases family 17
LOC_Os01g71860	Glycosyl hydrolases family 17, putative
LOC_Os01g71930	Glycosyl hydrolases family 17, putative
LOC_Os02g10660	Glycosyl hydrolases family 17, putative
LOC_Os02g53200	Glycosyl hydrolases family 17, putative
LOC_Os03g12140	Glycosyl hydrolases family 17, putative
LOC_Os03g12620	Glycosyl hydrolases family 17, putative
LOC_Os03g14210	Glycosyl hydrolases family 17, putative
LOC_Os06g04080	Glycosyl hydrolases family 17, putative
LOC_Os06g34020	Glycosyl hydrolases family 17, putative
LOC_Os06g39060	Glycosyl hydrolases family 17, putative
LOC_Os07g07340	Glycosyl hydrolases family 17, putative
LOC_Os07g13580	Glycosyl hydrolases family 17, putative
LOC_Os09g32550	Glycosyl hydrolases family 17, putative
LOC_Os04g27980	Glycosyl hydrolases family 18
LOC_Os07g43820	Glycosyl hydrolases family 18
LOC_Os01g39830	Glycosyl hydrolases family 35
LOC_Os06g42310	Glycosyl hydrolases family 35, putative
LOC_Os01g52710	Glycosyl transferase family 8, putative
LOC_Os02g50600	Glycosyl transferase family 8, putative
LOC_Os03g11330	Glycosyl transferase family 8, putative
LOC_Os03g18890	Glycosyl transferase family 8, putative
LOC_Os06g13760	Glycosyl transferase family 8, putative
LOC_Os07g45260	Glycosyl transferase family 8, putative
LOC_Os02g29530	glycosyltransferase quasimodo1 (ec 2.4.
LOC_Os01g34890	hypothetical protein
LOC_Os02g25630	hypothetical protein
LOC_Os03g07820	hypothetical protein
LOC_Os06g12460	hypothetical protein
LOC_Os08g33740	hypothetical protein
LOC_Os09g31270	hypothetical protein

LOC_Os10g28200	hypothetical protein
LOC_Os11g07090	hypothetical protein
LOC_Os01g08470	Leucine Rich Repeat, putative
LOC_Os01g41120	Leucine Rich Repeat, putative
LOC_Os06g49100	Leucine Rich Repeat, putative
LOC_Os07g07990	Leucine Rich Repeat, putative
LOC_Os03g16980	NAD dependent epimerase/dehydratase fam
LOC_Os06g08810	NAD dependent epimerase/dehydratase fam
LOC_Os06g44260	NAD dependent epimerase/dehydratase fam
LOC_Os07g47700	NAD dependent epimerase/dehydratase fam
LOC_Os09g32670	NAD dependent epimerase/dehydratase fam
LOC_Os11g37890	NAD dependent epimerase/dehydratase fam
LOC_Os02g54890	nucleotide sugar epimerase-like protein
LOC_Os06g05260	pectate lyase (EC 4.2.2.2) - maize
LOC_Os02g12300	pectate lyase precursor (ec 4.2.2.2)
LOC_Os01g21630	Pectinacetylesterase
LOC_Os01g66840	Pectinacetylesterase
LOC_Os01g66850	Pectinacetylesterase
LOC_Os01g44340	Pectinesterase
LOC_Os09g26360	pectinesterase (EC 3.1.1.11) [imported]
LOC_Os01g13320	Pectinesterase, putative
LOC_Os02g18650	Pectinesterase, putative
LOC_Os02g54190	Pectinesterase, putative
LOC_Os03g18860	Pectinesterase, putative
LOC_Os04g54850	Pectinesterase, putative
LOC_Os05g44600	Pectinesterase, putative
LOC_Os07g47830	Pectinesterase, putative
LOC_Os07g49100	Pectinesterase, putative
LOC_Os08g34900	Pectinesterase, putative
LOC_Os11g08750	Pectinesterase, putative
LOC_Os01g16770	Pollen allergen, putative
LOC_Os03g04020	Pollen allergen, putative
LOC_Os03g06020	Pollen allergen, putative
LOC_Os03g06050	Pollen allergen, putative
LOC_Os04g49410	Pollen allergen, putative
LOC_Os06g01920	Pollen allergen, putative
LOC_Os06g50960	Pollen allergen, putative
LOC_Os12g36040	Pollen allergen, putative
LOC_Os02g10300	polygalacturonase
LOC_Os06g40890	polygalacturonase - maize
LOC_Os01g43490	Polygalacturonase (pectinase)
LOC_Os11g14410	Polygalacturonase (pectinase)
LOC_Os01g44970	Polygalacturonase (pectinase), putative

LOC_Os05g50260	Polygalacturonase (pectinase), putative
LOC_Os06g01760	Polygalacturonase (pectinase), putative
LOC_Os06g31270	polygalacturonase homolog T9A21.20 - Ar
LOC_Os06g28670	polygalacturonase PG2
LOC_Os02g03750	polygalacturonase; 18642-16492, putativ
LOC_Os06g22980	probable cellulose synthase [imported]
LOC_Os03g18820	probable glycosyltransferase [imported]
LOC_Os12g05380	probable glycosyltransferase [imported]
LOC_Os06g38510	probable pectate lyase (EC 4.2.2.2) - a
LOC_Os06g09340	probable pectinesterase At2g26440 [impo
LOC_Os03g45390	putative beta-1,3-glucanase
LOC_Os03g58920	putative galactosyltransferase, 3'-part
LOC_Os03g25790	putative glucanase
LOC_Os03g28090	putative pectin methylesterase
LOC_Os11g03940	retrotransposon protein, putative, Ty1-
LOC_Os03g05360	retrotransposon protein, putative, uncl
LOC_Os11g08870	retrotransposon protein, putative, uncl
LOC_Os03g40270	reversibly glycosylated polypeptide
LOC_Os03g18520	Similar to beta-1,3-glucanase
LOC_Os05g35360	Similar to beta-galactosidase, putative
LOC_Os09g39920	Similar to cellulose synthase-like prot
LOC_Os12g35710	Similar to extensin-like protein - maiz
LOC_Os02g17600	Similar to galactoside 2-alpha-l-fucosy
LOC_Os09g37360	Similar to pectinesterase (EC 3.1.1.11)
LOC_Os09g39760	Similar to probable pectinesterase [imp
LOC_Os01g15910	Similar to utp--glucose-1-phosphate uri
LOC_Os06g25010	Similar to xylanase inhibitor protein I
LOC_Os01g71890	transposon protein, putative, CACTA, En
LOC_Os02g04540	transposon protein, putative, CACTA, En
LOC_Os08g03570	UDP-glucose 4-epimerase
LOC_Os09g35800	UDP-glucose 4-epimerase
LOC_Os04g52730	UDP-glucose 4-epimerase, putative
LOC_Os03g31210	UDP-glucose dehydrogenase
LOC_Os09g38030	UTP--glucose-1-phosphate uridylyltransf
LOC_Os02g17880	Xet3 protein
LOC_Os02g32750	xyloglucan 6-xylosyltransferase (ec 2.4
LOC_Os06g48200	xyloglucan endo-1,4-beta-D-glucanase (E
LOC_Os06g13040	xyloglucan endotransglucosylase/hydrola
LOC_Os04g51510	xyloglucan endotransglycosylase
LOC_Os06g48180	xyloglucan endotransglycosylase homolog
LOC_Os04g53950	xyloglucan endotransglycosylase, putati
LOC_Os02g52630	Xyloglucan fucosyltransferase
LOC_Os06g10930	Xyloglucan fucosyltransferase

LOC_Os06g10970	Xyloglucan fucosyltransferase
LOC_Os06g10980	Xyloglucan fucosyltransferase
LOC_Os09g28460	Xyloglucan fucosyltransferase
LOC_Os06g10960	Xyloglucan fucosyltransferase, putative
