# Supplementary material for: Comparison of Four ChIP-Seq Analytical Algorithms Using Rice Endosperm H3K27 Trimethylation Profiling Data
Source: PLoS One. 2011 Sep 30;6(9):e25260. doi: 10.1371/journal.pone.0025260 (PMC3184143; doi:10.1371/journal.pone.0025260)
Supplement: Table S2 — FindPeaks program parameters. (PDF) [file pone.0025260.s002.pdf]

Table S2. FindPeaks program parameters

FindPeaks		
Name	Value	Description
dist_type	1 200	method to extend tags
minimum	8	minimum number of tags for enrichment
hist_size	100	number of cells in the output FDR histogram
eff_size	3.66E8	mappable size of genome
trim	.2	portion of either side of peak to remove when calling peaks
subpeaks	.5	minimum relative valley depth to separate two peaks
mcfdr	20	iterations to use for Monte Carlo simulation

This table shows the parameters that were used when running FindPeaks. Default parameters were used for all other values.
