# Supplementary material for: Comparison of Four ChIP-Seq Analytical Algorithms Using Rice Endosperm H3K27 Trimethylation Profiling Data
Source: PLoS One. 2011 Sep 30;6(9):e25260. doi: 10.1371/journal.pone.0025260 (PMC3184143; doi:10.1371/journal.pone.0025260)
Supplement: Table S3 — USeq program parameters. (PDF) [file pone.0025260.s003.pdf]

Table S3. USeq program parameters

USeq			
Script	Name	Value	Description
PeakShiftFinder	-w	12	window size
	-d	8	minimum normalized window score
ScanSeqs	-p	75	peak shift, average distance between + and – strand peaks
	-w	589	window size
	-f	na	filter windows with high control read count
	-g	4	standard deviations off median of control read count (wrong)
EnrichedRegionMaker	-i	1,2,4	score indices, QValFDR, EmpFDR, and Log2Ratio
	-s	20,13,1	thresholds for scores, -10Log10(FDR/p-val) where 13 = 5%, 20 = 1%, etc.

This table shows the parameters that were used when running USeq. Default parameters were used for all other values.
