# Supplementary material for: Comparison of Four ChIP-Seq Analytical Algorithms Using Rice Endosperm H3K27 Trimethylation Profiling Data
Source: PLoS One. 2011 Sep 30;6(9):e25260. doi: 10.1371/journal.pone.0025260 (PMC3184143; doi:10.1371/journal.pone.0025260)
Supplement: Table S4 — PeakSeq program parameters. (PDF) [file pone.0025260.s004.pdf]

Table S4. PeakSeq program parameters

PeakSeq			
Script	Name	Value	Description
compile.py	merlen	36	tag length
create_signal_map	dna_fragment_length	200	length of sheared fragments
score_hits	dna_fragment_length	200	length of sheared fragments
	window_size	1.0E6	window size for scoring
	max_threshold	100	maximum allowed threshold for mapped reads
	max_gap	200, 350, 589	allowable distance to merge peaks
	FDR_required	0.05	required FDR from simulations
	number_of_sims	10	number of simulations to run per window
filter_hits	dna_fragment_length	200	length of sheared fragments
	bin_size	1000	bin size for linear regression
	max_count	3	maximum number of tags allowed to start at the same position
	extended_region_size	2000	distance to count control tags
	Pf	1	portion of peaks to consider for adjusting control tag counts
adjust_and_select_hits	pval_threshold	0.05	p-value to determine significant peaks

This table shows the parameters that were used when running PeakSeq. Default parameters were used for all other values. PeakSeq was run three times using a different max_gap parameter each time. All other parameters were the same for each run.
