# Supplementary material for: Comparison of Four ChIP-Seq Analytical Algorithms Using Rice Endosperm H3K27 Trimethylation Profiling Data
Source: PLoS One. 2011 Sep 30;6(9):e25260. doi: 10.1371/journal.pone.0025260 (PMC3184143; doi:10.1371/journal.pone.0025260)
Supplement: Table S5 — MACS program parameters. (PDF) [file pone.0025260.s005.pdf]

Table S5. MACS program parameters

MACS		
Name	Value	Description
mfold	5 (5)	threshold for high quality peaks when determining *d*
gsize	3.66E8	effective genome size
tsize	36	tag length
bw	120	bandwidth (length of sheared fragments)
pvalue	0.05	p-value to determine significant peaks

This table shows the parameters that were used when running MACS. Default parameters were used for all other values.
