# Supplementary material for: Comparison of Four ChIP-Seq Analytical Algorithms Using Rice Endosperm H3K27 Trimethylation Profiling Data
Source: PLoS One. 2011 Sep 30;6(9):e25260. doi: 10.1371/journal.pone.0025260 (PMC3184143; doi:10.1371/journal.pone.0025260)
Supplement: Table S6 — The primers used in semi-quantification PCR reactions. (PDF) [file pone.0025260.s006.pdf]

Table S6. The primers used in semi-quantification PCR reactions.

Locus	Forward Primer	Reverse Primer
LOC_Os01g04800	GTCAGCTTCTCCTCGACTTC	GAGTCAACTCCCTTCACCAC
LOC_Os01g18440	TGGCTCGCAAGAAGATCG	CTCCCCGTACACCACCAG
LOC_Os01g18584	GATCAAGGAGGTGGACTTCT	TTCAGCTACTCTAGTCCAACG
LOC_Os02g07430	AGGTGACATTCTCGAAGAGG	GCAGTACTCGAACATCTTGC
LOC_Os02g45850	AAAGAAGAGGAGGACGAGGA	GTCGAACATGTGCTCCTTC
LOC_Os03g63810	AGATCACCATCCCTTACCA	GTGAGGTGGTGGTATGAGTC
LOC_Os04g41229	AGCACCATTTCCATCAGTTC	GAGTGCAGTAACCGTTGTCC
LOC_Os05g11130	GAGACGCTGCAGTTCATCT	CCGTACCTACGGAATCAATC
LOC_Os05g20930	CCAGCAAGGTACTGGATCT	CTCCCATGACTCGTAGTAGG
LOC_Os05g48990	CTCCTTGCTCTCCTCAGGTA	TCACACCCTGCAAATTACAC
LOC_Os06g11330	AGTTCGCCAGCTCCAAGTA	CGAGCGGATCGAAAACTA
LOC_Os07g13260	TCAACAACTACTCCCTCACC	CTTGGAGTTAGAGGGCTTG
LOC_Os08g02160	GATCATCATCAGGATGTCGT	GGAGGTAGAACTCGATGAGC
LOC_Os08g06370	AGCAAAGTGAGATCAAGCTG	AAAGAACCTCGCGTTGAG
LOC_Os09g24490	GTTGGGTGAGCTAGCGATGT	GTCGACACACCCTCCGTATC
LOC_Os10g39130	ATACCTTCTTCTCGTTCTCG	ACCCGATTACCACAGAGAG
LOC_Os11g29870	CACTTCATGAGCTCCTCTTC	GAGAGCTCCTCCATCCTAAT
LOC_Os12g10540	GGGCAGGATTGAGATCAAGA	GGCTGGAGAAGACGATGAGA
LOC_Os03g09930	CAAGCCTGTGGAATGATCTC	TGTAGGTTTTCCACCAGTCA
LOC_Os07g40570	CAGATCACAGCCTGATTACG	CATCATGCTCATCCTGGTTA
LOC_Os12g43640	GCTGTTCTACAATGGCTTCAC	GACACGTCGATGAAGTTGAA
LOC_Os01g10504	GTCGATGACGGAGCTGACC	GTTCGTCGTGTTCTCGATCC
LOC_Os12g44380	GTTCTCACCGTCGGATTCT	GTCCCTGTGTAGCGTTGTT
